# Clustering of asymptomatic *Plasmodium falciparum* infection and the effectiveness of targeted malaria control measures

**DOI:** 10.1186/s12936-019-3063-9

**Published:** 2020-01-21

**Authors:** Jeffrey G. Shaffer, Mahamoudou B. Touré, Nafomon Sogoba, Seydou O. Doumbia, Jules F. Gomis, Mouhamadou Ndiaye, Daouda Ndiaye, Ayouba Diarra, Ismaela Abubakar, Abdullahi Ahmad, Muna Affara, Davis Nwakanma, Mary Lukowski, James C. Welty, Frances J. Mather, Joseph Keating, Donald J. Krogstad

**Affiliations:** 1 School of Public Health and Tropical Medicine, Tulane University, 1440 Canal Street, New Orleans, LA 70112 USA; 2 University of Sciences, Techniques and Technologies of Bamako, Bamako, Mali; 3 Cheikh Anta Diop University, Dakar, Senegal; 4 Medical Research Council Units, Fajara and Basse, Serekunda, The Gambia; 5 ScienceTRAX, Macon, GA USA

**Keywords:** ArcGIS system for working with maps and geographic data (Esri—Redlands, CA), Clustering of *P. falciparum* infection in space, time or both space and time, Geographic information system (GIS), Global positioning system (GPS), Mali, *Plasmodium falciparum*, SaTScan software to analyse spatial, temporal and spatio-temporal data (Boston, MA), Spatial Scan Statistic to determine whether a set of points is distributed randomly or has clusters or clustering, Senegal, The Gambia, World Geodetic System 1984 (WGS) reference coordinate system for GPS

## Abstract

**Background:**

Because clustering of *Plasmodium falciparum* infection had been noted previously, the clustering of infection was examined at four field sites in West Africa: Dangassa and Dioro in Mali, Gambissara in The Gambia and Madina Fall in Senegal.

**Methods:**

Clustering of infection was defined by the percent of persons with positive slides for asexual *P. falciparum* sleeping in a house which had been geopositioned. Data from each site were then tested for spatial, temporal and spatio-temporal clustering in relation to the prevalence of infection from smear surveys.

**Results:**

These studies suggest that clustering of *P. falciparum* infection also affects the effectiveness of control interventions. For example, the clustering of infection in Madina Fall disappeared in 2014–2016 after vector control eliminated the only breeding site in 2013. In contrast, the temporal clustering of infection in Dioro (rainy season of 2014, dry season of 2015) was consistent with the loss of funding for Dioro in the second quarter of 2014 and disappeared when funds again became available in late 2015. The clustering of infection in rural (western) areas of Gambissara was consistent with known rural–urban differences in the prevalence of infection and with the thatched roofs, open eaves and mud walls of houses in rural Gambissara. In contrast, the most intense transmission was in Dangassa, where the only encouraging observation was a lower prevalence of infection in the dry season. Taken together, these results suggest: (a) the transmission of infection was stopped in Madina Fall by eliminating the only known breeding site, (b) the prevalence of infection was reduced in Dioro after financial support became available again for malaria control in the second half of 2015, (c) improvements in housing should improve malaria control by reducing the number of vectors in rural communities such as western Gambissara, and (d) beginning malaria control during the dry season may reduce transmission in hyperendemic areas such as Dangassa.

**Conclusions:**

From a conceptual perspective, testing for spatial, temporal and spatio-temporal clustering based on epidemiologic data permits the generation of hypotheses for the clustering observed and the testing of candidate interventions to confirm or refute those hypotheses.

## Background

With 60% of the global total, sub-Saharan Africa has the world’s greatest malaria burden [1, 2]. *Plasmodium falciparum* is the predominant parasite and causes most cases [3]. Strategies used most frequently to reduce *P. falciparum* infection and malarial disease include: (i) insecticide-impregnated bed nets (ITNs) to reduce vector-borne transmission, (ii) intermittent preventive treatment with pyrimethamine-sulfadoxine in the second and third trimesters of pregnancy (IPTp) to reduce placental malaria and its neonatal consequences, (iii) treatment of malaria with artemisinin-based combination therapy (ACT) to clear infections rapidly, and may also include (iv) seasonal malaria chemoprevention (SMC) for children less than 5 years of age [4].

Spatial clustering of malaria has been suggested in a number of studies, most frequently with *P. falciparum* infection. Heterogeneity in spatial and temporal distributions of *P. falciparum* infection has been identified in communities with both hypoendemic and intense transmission and in communities close to sea level as well as highland communities at more than 1800 m [5]. In addition, hotspots with an increased prevalence of infection and incidence of disease have been identified in Dielmo, Senegal [6] and near the Yamé River in Bandiagara, Mali [7]. Although a study of the peri-urban settlement of Sotuba on the Niger River near Bamako did not reveal clustering of *P. falciparum* infection in the rainy season, it did reveal clustering (an increased prevalence of *P. falciparum* infection) in the dry season that was partially explained by decreased use of ITNs [8]. Gaudart et al. found a downward temporal trend in *P. falciparum* parasitemia among children less than 12 years of age in Bancoumana, Mali and detected six spatio-temporal clusters based on 22 smear surveys from 1996 to 2001 [9]. Stresman et al. have noted that within-village malaria transmission may be concentrated within a small proportion of high burden households in The Gambia [10]. Outside West Africa, other plasmodial species have also shown spatial clustering. For example, Rosas-Aguirre et al. have reported that the prevalence of *Plasmodium vivax* infection is highly heterogeneous on the northwestern coast of Peru [11].

Previous studies have also linked wall materials and lack of ITN use to the clustering of *P. falciparum* infection [12, 13]. Mosha et al. suggested large-scale patterns of transmission may be driven by climate and ecology, whereas hotspots may be driven by variations in household and micro-environmental factors [14]. *Plasmodium falciparum* infection is reported to cluster in areas where transmission is intense [12]. Malaria hotspots may maintain the transmission of *P. falciparum* infection in low transmission seasons and have been suggested as a driving force in the high transmission (rainy) season [10, 14].

Greenwood [15] has noted that local variations in the epidemiology of malaria must be accounted for in planning malaria control interventions. Mlacha et al. also noted the potential value of information from spatial studies for the targeted delivery of interventions [16], and suggested that heterogeneity of malaria transmission within villages may be present long before the pre-elimination phase. They suggest that identifying and targeting hotspots of malaria transmission should be a cornerstone for both malaria control and its elimination [10, 12]. In addition, Landier et al. have noted that spatio-temporal analysis of the clustering of *P. falciparum* infection may be necessary to guide decision-making for malaria control [17].

The West African International Centers of Excellence for Malaria Research (ICEMRs) perform studies to identify and resolve the most important obstacles to malaria control [18]. From 2012 to 2016, the West African ICEMRs performed longitudinal cohort studies at study sites in Mali (n = 2), The Gambia (n = 1) and Senegal (n = 1) which previously had high levels of plasmodial infection and malarial disease. For example, the estimated annual incidence rates for uncomplicated malaria in Mali, The Gambia and Senegal in 2015 were 448.6, 208.8, and 97.6 cases/year/1000 persons [18, 19].

This manuscript uses geographic information system (GIS) data and cluster analysis to test for spatial, temporal and spatio-temporal clustering of *P. falciparum* infection. The rationale for this approach is the hypothesis that there should be logical links between the clustering of *P. falciparum* infection and the optimal malaria control interventions in endemic areas such as West Africa.

## Methods

### Study sites

**Fig. 1:**
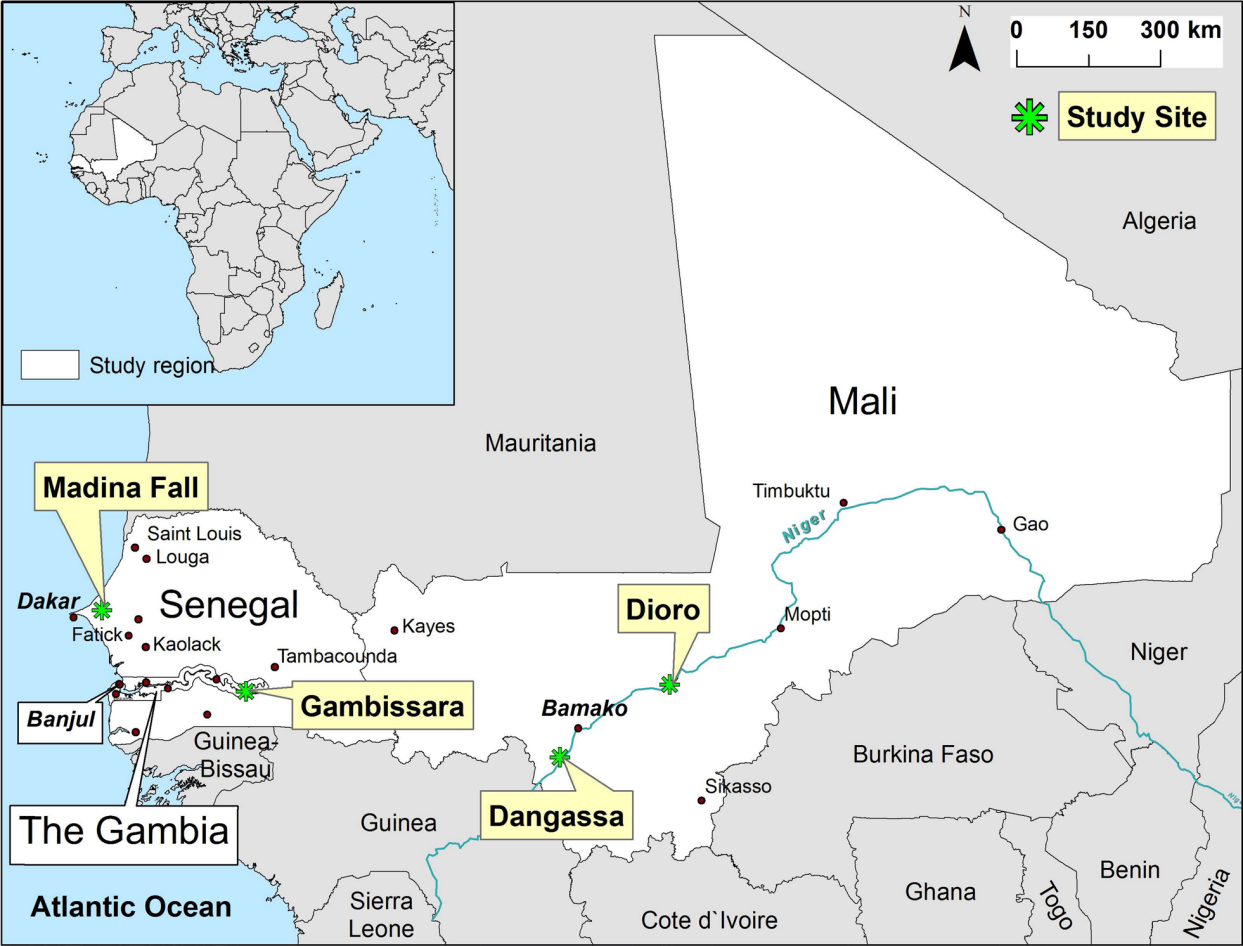
Map with West African study sites in The Gambia, Senegal and Mali. The purpose of this map is to localize the four West African study sites. Traveling west from the Atlantic Ocean in Senegal, the first study site is Madina Fall (a peri-urban site in Thiès, Senegal) 70 km east of Dakar, which is indicated by an asterisk on the map for the name of the study site (Madina Fall). Gambissara in The Gambia is southeast of Madina Fall and 310 km east of Banjul at the eastern end (Upper River Region) of the Gambia River and is also marked by an asterisk. The two Mali study sites are both on the Niger River which flows to the east for 3360 km after leaving Bamako on its way to southeast Nigeria (Port Harcourt). Dangassa is 70 km south of Bamako in the savannah region of Mali and has extensive seasonal rainfall. In contrast, Dioro is 290 km northeast of Bamako in the much drier Sahelian region of Mali. As with Madina Fall and Gambissara, Dangassa and Dioro are also marked by asterisks

This study examined four communities in Mali, Senegal and The Gambia. The study sites in Mali were the villages of Dangassa and Dioro: Dangassa is a rural community in southwest Mali on the Niger River 100 km southwest of Bamako and 160 km north of the Guinea border with year-round malaria transmission [20]. In contrast, Dioro is a rural community in the Segou Region of Mali on the Niger River at the southern edge of the Sahel which is 290 km northeast of Bamako and has primarily seasonal transmission (October–December in the rainy season) [21]. The study site in The Gambia was Gambissara, a rural community in the Upper River Region of The Gambia which is 10–15 km north of the Senegal border and 311 km east of Banjul [22]. The Senegal study site was Madina Fall [23], a peri-urban community next to the south-central city of Thiés 70 km east of Dakar which has limited transmission and a low year-round prevalence of *P. falciparum* infection (1–2%; Table 1, Fig. 1).

**Table 1:** Seasonal (temporal) clustering of *P. falciparum* infection at the household level at four West African study sites in Mali (n = 2), Senegal (n = 1) and The Gambia (n = 1)

Study site(s)	Location of study site (based on GPS)		Mean rainy season prevalence of infection		Mean dry season prevalence of infection		Rainy vs. dry season (*t*-test)	# houses selected	Annual rainfall
	Latitude	Longitude	% Pos	(SD)	% Pos	(SD)	*p*-value	# Houses	mm
Dangassa, Mali	12.11 N	− 8.33 W	44.5	(22.5)	37.6	(19.0)	0.0003	239	1023
Dioro, Mali	13.45 N	− 6.27 W	31.5	(19.7)	17.0	(16.1)	< 0.0001	305	591
Gambissara, The Gambia	13.32 N	− 14.22 W	4.4	(10.4)	9.1	(14.6)	0.002	141	1038
Madina Fall, Senegal	14.81 N	− 16.92 W	0.4	(1.9)	1.0	(4.6)	0.146	146	503
Totals								Ʃ 831	Ʃ = 2705

GPS coordinates (latitude and longitude) are provided for each study site in columns 2 and 3, mean household prevalences of infection (percent of positive thick smears for each household) are in columns 4 and 6 and the standard deviations for those prevalences are in columns 5 and 7 (for the rainy and dry seasons, respectively). The numbers of households selected randomly from these sites which agreed to participate in this study are listed in column 9 for each study site and the annual rainfall totals for each site are listed in column 10. Testing to determine whether prevalences of infection were different in the dry vs. wet seasons was performed using the GraphPad QuickCalcs *t*-test calculator (https://www.graphpad.com/quackcalcs/ttest1/); the results of that testing are in column 8

### Study design and data collection

At each study site, households (single family units that lived and cooked together) were selected randomly for participation, to establish four rolling epidemiologic cohorts of study subjects. It is worth mentioning that, in this area of West Africa, the Sarahouli households in Gambissara are often much larger than those of other ethnic groups. Data collection was carried out biannually using thick smear surveys and questionnaires which also examined household factors and obtained geographic coordinates for the houses studied using stand-alone Global Positioning System (GPS) devices (Trimble, Inc.—Sunnyvale, CA). However, there was only one (rainy season) visit to each participating household in The Gambia in 2012 and only one (dry season) visit to each participating household in Mali (Dangassa, Dioro) in 2013. Data from these surveys were used to estimate the prevalence of *P. falciparum* infection in participating households at each study site (based on asexual malaria parasite counts performed with oil immersion magnification at the field site laboratories). Those data were adjusted by dividing the number of subjects with positive smears for asexual *P. falciparum* parasites by the total number of persons tested from that household ([# positive/# tested per house] × 100 = % infected).

### Inclusion criteria

Participants in the twice-yearly smear surveys were required to have a study identification number (as evidence of written informed consent for their participation), one or more blood smear results from the twice-yearly smear surveys and to sleep at night in a study household for which GPS coordinates were available. Study identification numbers were issued only to persons who had provided informed consent or assent for their participation. Household members who did not have study identification numbers were not permitted to participate in the study or counted in the denominators used to estimate the prevalence of *P. falciparum* infection. GPS coordinates were obtained for each household using two GPS devices (Trimble Nomad—Sunnyvale, CA), one of which was designated the “base” unit and placed in a central location at the study site (after being geopositioned for 24 h). The other GPS unit, designated the “rover” GPS device, was used to take readings for 10–15 min at each household participating in the study. At the end of each day in the field, readings from the “base” and “rover” GPS units were downloaded to a laptop and used to geoposition the households participating in the study.

### Classification of transmission seasons

Transmission seasons varied across study sites and were classified as “dry” or “rainy” [20–23]. For the Mali sites, seasons were classified as dry (January 1–June 30) and rainy (July 1–December 31) [20, 21]; for The Gambia as dry (October 1–June 15) [22] and rainy (June 16–September 30) and for Senegal as dry (December 1–April 30) and rainy (May 1–November 30) [23].

### Statistical analyses

Results were expressed as correlation coefficients, *p*-values, frequencies, percentages, means and standard deviations. The Spatial Scan Statistic was generated using the SaTScan application (version 9.6) to examine both circular and non-circular clustering patterns [24, 25]. Because one smear survey was not performed at Gambissara in 2012 or at the Mali sites in 2013, survey results were stratified by season. The Statistical Analysis System (SAS Institute—Cary, NC, version 9.4) was used for data linkage, management and analysis. Distances between households were estimated using the ArcGIS (Esri—Redlands, CA, v. 10.1.3) Average Nearest Neighbor tool. For analyses using the Stata Geonear application (StataCorp—College Station, TX), a normal probability model was used to identify clusters of households with a low or high prevalence of *P. falciparum* infection. Additional settings for the SaTScan analyses included minimum and maximum time windows of 1 and 3 years, a maximum spatial cluster size set at 25% of the households for each site and no geographic overlap. Fisher’s Exact Test was used for categorical comparisons and the Wilcoxon test to compare group means. Random effects models based on the SAS Glimmix Procedure were used to assess the relationship between household-level factors such as reported bed net use and the presence of households in a cluster. Statistical significance levels were 5% (*p *= 0.05) and statistical tests on multiple household factors were corrected for multiple testing using the Bonferroni correction.

### Mapping of households with *P. falciparum* infection

Geocoordinates were captured for each sampled household using portable global positioning systems in World Geodetic System (WGS) 1984 format. GPS data were linked with household prevalence data and mapped using ArcGIS (Esri—Redlands, CA, v 10.1.3). Satellite images were obtained from the pre-loaded base maps in ArcGIS. Maps simultaneously displaying the four study sites (Fig. 1) were projected using the WGS 1984 World Mercator projection. Maps for the Dangassa and Dioro study sites were projected using the WGS 1984 Universal Transverse Mercator (UTM) Zone 30 North (N) coordinate system; maps for Senegal and The Gambia study sites were projected using the WGS 1984 UTM Zone 28 N coordinate system. Because of the small size and large scale of these maps, visual differences due to projecting to the WGS 1984 geographic coordinate systems were negligible.

### Definition of clusters

Spatial clustering was defined as groups of households with lower or higher prevalence of *P. falciparum* infection than expected based on the null hypothesis for the spatial randomness of *P. falciparum* infection. Clusters with a high prevalence of *P. falciparum* infection were examined with the Spatial Scan Statistic to compare the mean prevalence of infection inside the cluster to the mean prevalence of infection outside the cluster. Spatio-temporal cluster analyses were also performed using the Spatial Scan Statistic, which detects two-dimensional clusters with cylindrical shapes, in which the height of the cylinder represents time and the circular ends of the cylinder represent the spatial cluster(s) identified by the Spatial Scan Statistic. The null hypothesis for the spatio-temporal approach is the spatial randomness of *P. falciparum* infection in both space and time. These data were considered a time series because this was a cohort-based longitudinal study in which the same subjects were sampled repetitively over time. Clusters of single households were considered spatial outliers.

## Results

This study included 6014 subjects at 4 study sites: Dangassa, Mali, n = 1317; Dioro, Mali, n = 1481; Gambissara, The Gambia, n = 1380 and Madina Fall, Senegal, n = 1836 (Fig. 1). Data for individual subjects were linked to the 831 households enrolled across the 4 study sites: Dangassa, n = 239; Dioro, n = 305; Gambissara, n = 141 and Madina Fall, n = 146 (Table 1).

The four study sites differ with respect to virtually all household characteristics, except the sale or distribution of mosquito nets, which was uncommon at all sites (< 2%). Average household sizes were greater in Gambissara (24.30 persons per household) than at the other 3 sites (7.84, 7.45 and 11.30 persons per household, for Dangassa, Dioro, and Senegal, respectively; *p *< 0.001).

### Spatial clustering of *P. falciparum* infection

#### Gambissara, The Gambia

**Fig. 2:**
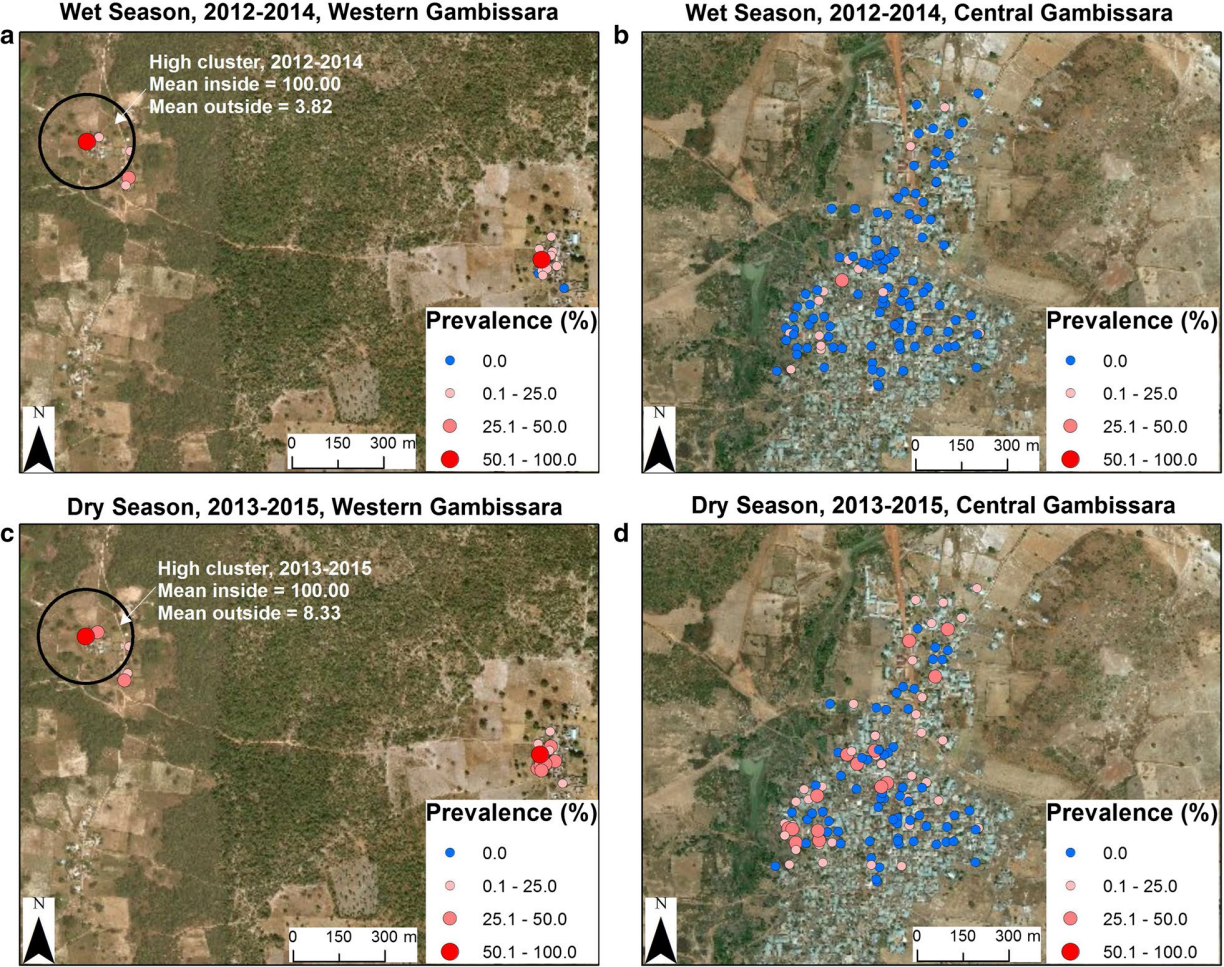
Gambissara, The Gambia: spatial clustering of *P. falciparum* infection. **a**, **b** Rainy Seasons, 2012–2014. Household-level prevalence of *P. falciparum* infection was low (not detectable using the color-coded scale in the legend) in Gambissara Town, but readily detectable in the more rural area of western Gambissara. **a** for the western (rural) area of Gambissara in the rainy season is on the left side of Fig. 2; **b** Gambissara Town during the rainy season is on the right side of Fig. 2. **c**, **d** Dry Seasons, 2013–2015. As in the rainy season, the household-level prevalence of *P. falciparum* infection was undetectable in Gambissara Town. In contrast, households with *P. falciparum* infection were readily detectable in the more rural area of western Gambissara where housing is less secure and thatched roofs and mud walls provide less protection from anopheline vectors. **c** The western (rural) area of Gambissara in the dry season is on the left side of Fig. 2; **d** Gambissara Town in the dry season where better housing provided greater protection against *Anopheles gambiae* is on the right side of Fig. 2. Source and service layer credits for satellite imagery: Esri, DigitalGlobe, GeoEye, Earthstar, Geographics, CNES/Airbus DS, USDA, USGS, AEX, Getmapping, Aerogrid, IGN, IGP, swisstopo, and the GIS User Community

Spatial clustering of *P. falciparum* infection (household-level prevalence of infection) was observed in The Gambia during both the dry and rainy seasons in the rural (western) area of Gambissara where housing was less secure in terms of malaria prevention (thatched roofs, open eaves, mud walls) [26–28], but not in the more urban area of Gambissara where *P. falciparum* infection was less frequent (Fig. 2, metal roofs, closed eaves, brick walls). These differences in the clustering of *P. falciparum* infection between rural and urban areas of Gambissara were present during both the dry and rainy seasons from 2012 to 2016 (Table 1, Fig. 2).

#### Dangassa and Dioro in Mali

The prevalence of *P. falciparum* infection during the study period is shown in Table 1. The prevalence of *P. falciparum* infection was frequently over 50% in Dangassa and over 30% in Dioro (top quartiles of ≥ 51.5% and ≥ 33.4% for Dangassa and Dioro, respectively). Although the densities (inter-household distances) of the houses sampled were less in Dioro (21.28 m *vs.* 28.50, 30.14 and 30.33 for Dangassa, Dioro and Madina Fall, *p *< 0.001), the magnitude of those differences was modest (7, 9 and 9 m, respectively).

When the clustering of *P. falciparum* infection was examined using the Spatial Scan Statistic, it revealed clustering only at the Dangassa and Gambissara study sites. Circular clusters identified at the Dangassa site included two low prevalence clusters: one in the dry season and one in the rainy season (Fig. 4c and e). However, no spatial clustering of *P. falciparum* infection was identified in Dioro or Senegal.

### Temporal clustering of *P. falciparum* infection

#### Dioro, Mali

**Fig. 3:**
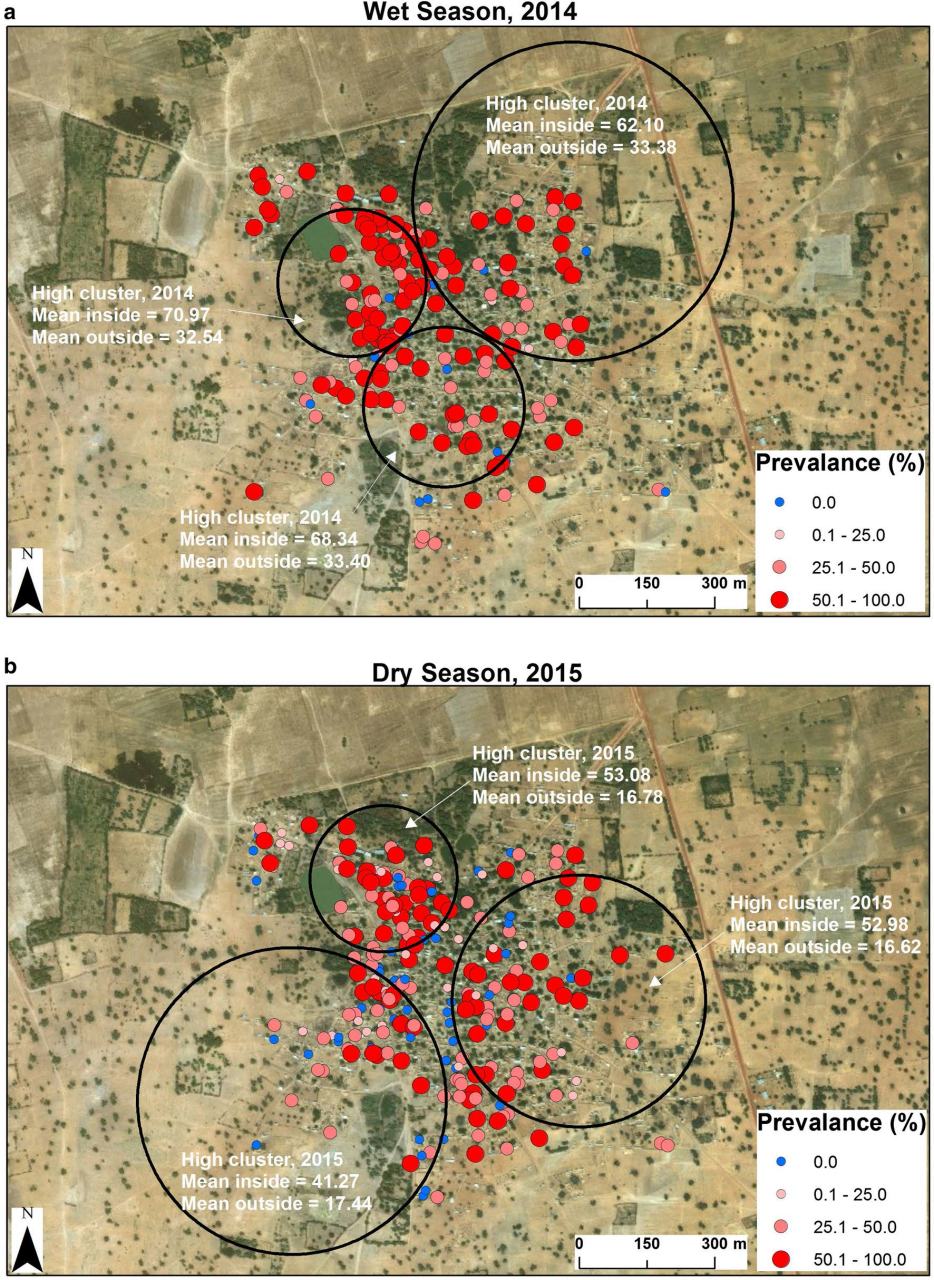
Dioro, Mali: temporal clustering of *P. falciparum* infection. **a** Rainy Season of 2014. During the dry season in 2014 (March–June), the funds which had been provided to the Millennium Villages Project in Dioro ran out. As a result, the financial support available previously to reduce transmission (e.g., maintenance and repair of ITNs, ACTs for children with malarial disease, IPT for pregnant women) was no longer available and the prevalence of *P. falciparum* infection rose from 8 to 10% in previous years to 60% by October 2014 during the 2014 rainy season (**a**). **b** Dry Season of 2015. In the absence of other financial support, the prevalence of *P. falciparum* infection remained high during the dry season of 2015 (January–June). Source and service layer credits for satellite imagery: Esri, DigitalGlobe, GeoEye, Earthstar, Geographics, CNES/Airbus DS, USDA, USGS, AEX, Getmapping, Aerogrid, IGN, IGP, swisstopo, and the GIS User Community

Temporal clustering was found at all 4 study sites in the dry and rainy seasons, except for Senegal (Madina Fall) in the rainy season (Table 2). The most marked example of temporal clustering was at the Dioro site in Mali when support was no longer available for: (1) repair and replacement of ITNs, (2) ACT treatment of acute malaria or (3) IPTp for pregnant women beginning in May–June of 2014. By October 2014, the prevalence of *P. falciparum* in the community had risen to 60% (Fig. 3a) and remained high through the spring (dry season) of 2015 (Fig. 3b).

**Table 2:** Clusters of high and low prevalences of *P. falciparum* infection detected using the spatial scan statistic

Spatial clustering by year and season				$\overline{\text{x}}$ inside cluster	$\overline{\text{x}}$ outside cluster	Cluster size (km)	Std dev of clusters	*p*-value
	Years	# HH’s	Cases					
Dangassa	2012–16	32	239	30.01	43.20	0.13	31.52	0.001
Dioro	2012–16	1	3	100.0	26.70	0.00	30.99	0.159
Gambissara	2012–16	1	6	100.0	6.12	0.00	12.07	0.001
Madina Fall	2012–16	1	2	25.00	0.48	0.00	3.30	0.021

Spatial clustering by year and season				$\overline{\text{x}}$ inside cluster	$\overline{\text{x}}$ outside cluster	Cluster size (km)	Std dev of clusters	*p*-value
Dry season	Years	# HH’s	Cases					
Dangassa	2012–16	32	110	24.57	39.00	0.13	30.44	0.009
Dioro	2012–16	1	2	100.0	18.61	0.00	27.74	0.240
Gambissara	2012–16	16	72	27.39	6.03	1.52	14.12	0.001
Madina fall	2012–16	3	9	11.11	0.55	0.08	4.35	0.284

Spatial clustering by year and season				$\overline{\text{x}}$ inside cluster	$\overline{\text{x}}$ outside cluster	Cluster size (km)	Std dev of clusters	*p*-value
Wet season	Years	# HH’s	Cases					
Dangassa	2012–16	35	119	34.91	47.96	0.12	31.92	0.043
Dioro	2012–16	1	4	100.0	34.33	0.00	31.76	0.073
Gambissara	2012–16	1	3	100.0	3.82	0.00	8.56	0.001
Madina Fall	2012–16	1	4	6.25	0.28	0.00	2.10	0.284

Temporal clustering—year and season				$\overline{\text{x}}$ inside cluster	$\overline{\text{x}}$ outside cluster	Cluster size (km)	Std dev of clusters	*p*-value
Dry season	Years	# HH’s	Cases					
Dangassa	2013–2014	239	416	48.54	24.98	NA	28.49	0.001
Dioro	2015	305	238	43.38	10.68	NA	24.16	0.001
Gambissara	2014–2016	141	397	6.68	14.35	NA	15.48	0.001
Madina Fall	2013	146	144	2.02	0.12	NA	4.53	0.001

Temporal clustering—year and season				$\overline{\text{x}}$ inside cluster	$\overline{\text{x}}$ outside cluster	Cluster size (km)	Std dev of clusters	*p*-value
Wet season	Years	# HH’s	Cases					
Dangassa	2014–2015	239	353	55.82	37.58	NA	30.96	0.001
Dioro	2014	305	176	63.27	28.42	NA	29.13	0.001
Gambissara	2013–2014	141	267	5.26	3.42	NA	11.16	0.282
Madina Fall	2014	146	91	0.80	0.23	NA	2.16	0.097

Space/time clustering—year/season				$\overline{\text{x}}$ inside cluster	$\overline{\text{x}}$ outside cluster	Cluster size (km)	Std dev of clusters	*p*-value
Dry season	Years	# HH’s	Cases					
Dangassa	2014	56	50	66.08	35.14	0.92	29.92	0.001
Dioro	2015	81	57	52.98	16.62	0.28	26.63	0.001
Gambissara	2013–2015	1	3	100.00	8.33	0.00	14.34	0.001
Madina Fall	2013	3	3	33.33	0.55	0.08	3.68	0.049

Space/time clustering—year/season				$\overline{\text{x}}$ inside cluster	$\overline{\text{x}}$ outside cluster	Cluster size (km)	Std dev of clusters	p-value
Wet season	Years	# HH’s	Cases					
Dangassa	2016	60	43	21.07	47.41	0.61	31.70	0.002
Dioro	2014	67	53	70.97	32.54	0.16	30.84	0.001
Gambissara	2012–2014	1	3	100.00	3.82	0.00	8.56	0.001
Madina Fall	2013–2014	1	2	12.50	0.28	0.00	2.03	0.333

*HH* household; $\overline{\text{x}}$ mean value

The results provided were generated for the spatial, temporal and spatiotemporal analyses performed using the Spatial Scan Statistic. Each row represents the most likely cluster (based on the most significant *p*-value). Note that each row shows only the most likely cluster and that secondary clusters are not shown for brevity

### Spatio-temporal clustering of *P. falciparum* infection

#### Dangassa and Dioro, Mali; Gambissara, The Gambia and Madina Fall, Senegal

**Fig. 4:**
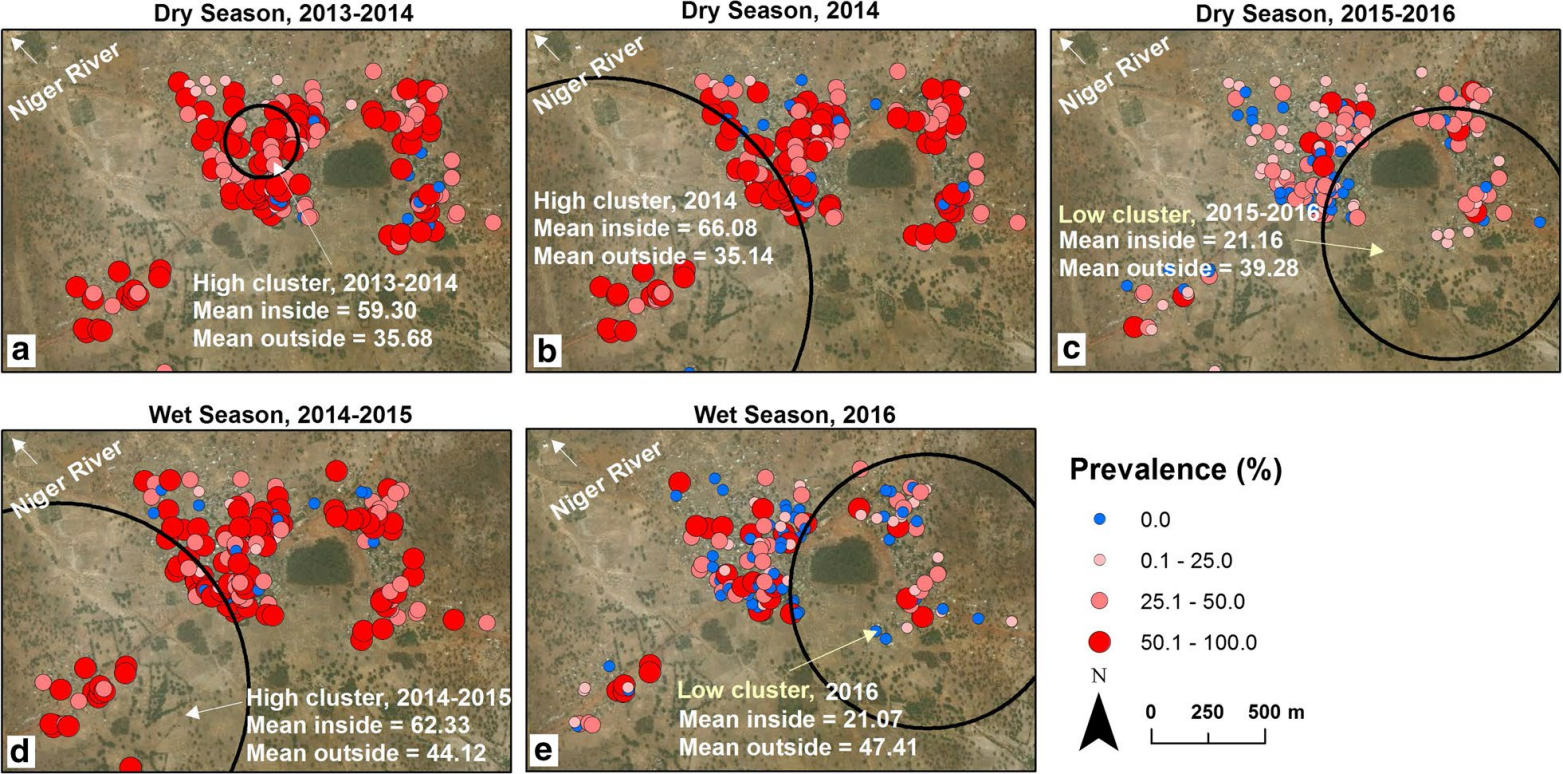
Dangassa, Mali: spatio-temporal clustering of *P. falciparum* infection. **a**–**c** Dry Seasons, 2013–2014, 2014 and 2015–2016. In both the 2013–2014 and 2014 dry seasons, the mean percent of positive smears in high clusters was 59–66% vs. 21% for low clusters. **d**, **e** Rainy Seasons, 2014–2015 and 2016. Similarly, in the rainy season, the mean percent of positive smears for high clusters was 62% vs. 21% for low clusters. Source and service layer credits for satellite imagery: Esri, DigitalGlobe, GeoEye, Earthstar, Geographics, CNES/Airbus DS, USDA, USGS, AEX, Getmapping, Aerogrid, IGN, IGP, swisstopo, and the GIS User Community

Large spatio-temporal clusters (with more than 50 households) of high and low *P. falciparum* prevalence households in space and time were identified at both the Dangassa and Dioro study sites. In contrast, spatio-temporal clustering was limited to small numbers of households at the Gambissara and Madina Fall study sites. At the Dangassa site, clusters with a high prevalence of *P. falciparum* infection occurred at similar locations during the dry season of 2014 (Fig. 4b) and the rainy seasons of 2014–2015 (Fig. 4d). In contrast, spatio-temporal clusters of households with a low prevalence of *P. falciparum* infection occurred during the dry season of 2015–2016 (Fig. 4c) and the rainy season of 2016 (Fig. 4e). During the dry season, a high prevalence cluster was observed in 2013–2014 in the western part of Dangassa, and one low prevalence cluster in 2015–2016 in the eastern part of Dangassa. During the rainy season, one high prevalence cluster was observed in western Dangassa during 2014–2015 (Fig. 4d) and a low prevalence cluster in eastern Dangassa during 2016 (Fig. 4e).

#### Dioro, Mali

In Dioro, there was an increase in the prevalence of *P. falciparum* infection during the rainy season of 2014 which continued through the 2015 dry season (Fig. 3). The spatio-temporal clusters with the greatest prevalence(s) of infection occurred during the rainy season of 2014 (means inside the clusters were 70.97%, 68.34% and 68.34%; means outside the clusters were 33.38%, 33.40% and 32.54%, *p *= 0.001). At the Dioro site, the high prevalence of *P. falciparum* infection during 2014 and 2015 occurred after a lapse in funding for malaria prevention and treatment because financial support was no longer available for the Millennium Villages Project.

#### Gambissara, The Gambia

Two spatio-temporal clusters of *P. falciparum* infection were observed in Gambissara for a single household with a 100% prevalence of infection in the dry seasons of 2013–2015 and the rainy seasons of 2012–2014. This was the only household with 100% smear positivity in the study and is in western Gambissara at the center of a rural area where the houses have thatched roofs, open eaves and mud walls and *P. falciparum* infection is more frequent than in Gambissara Town (Fig. 2).

#### Madina Fall, Senegal

**Fig. 5:**
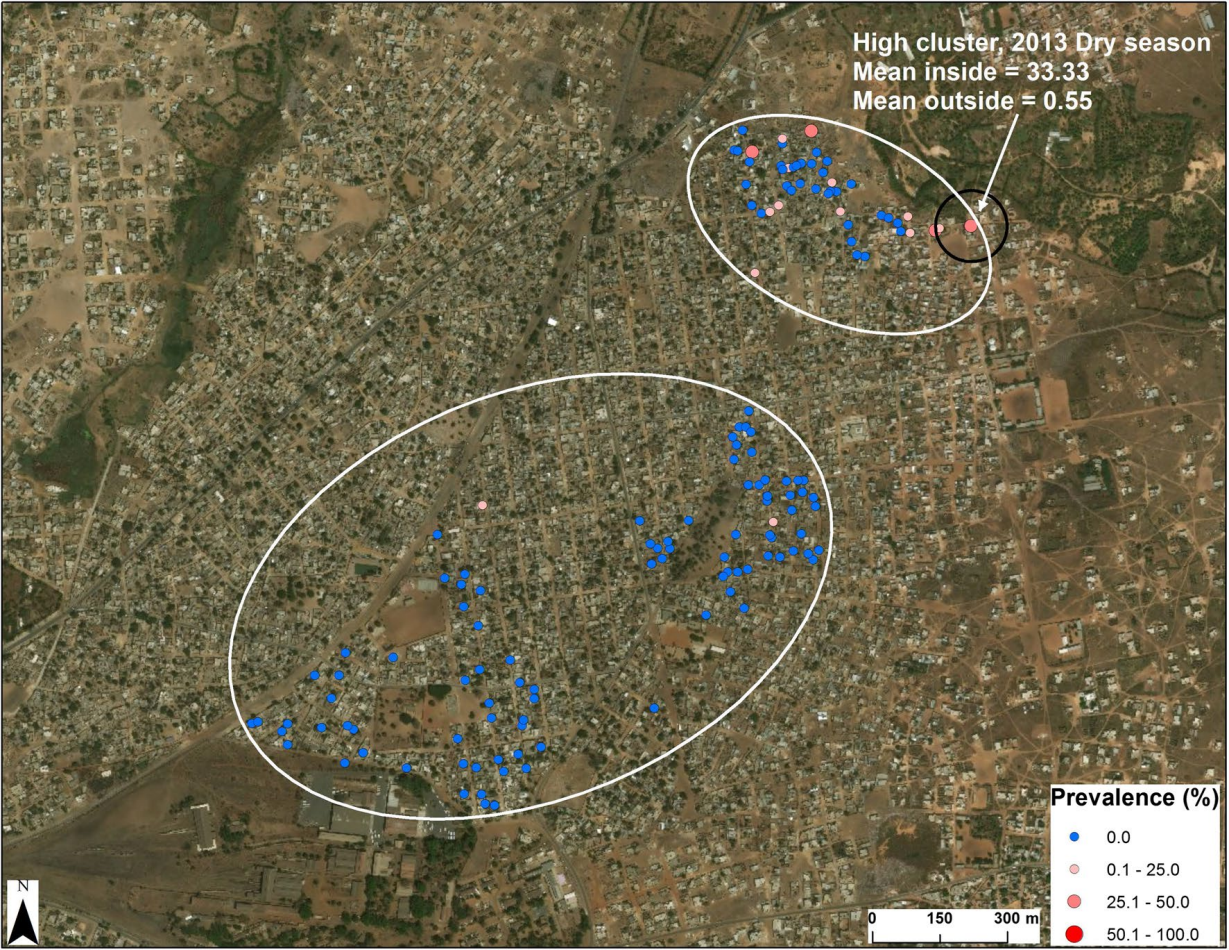
Spatio-temporal clustering of *P. falciparum* infection in Madina Fall. In the upper right-hand corner of this image, there is a circle for a high prevalence cluster of households with *P. falciparum* infection. As noted in the text, this cluster was next to the only breeding site in this community and disappeared as a focus of human infection after larviciding and basic vector control measures were implemented in 2013. Source and service layer credits for satellite imagery: Esri, DigitalGlobe, GeoEye, Earthstar, Geographics, CNES/Airbus DS, USDA, USGS, AEX, Getmapping, Aerogrid, IGN, IGP, swisstopo and the GIS User Community

In Madina Fall (Senegal) a single spatio-temporal cluster of 3 households was observed during the 2013 dry season (mean percent positive inside = 33.33, mean percent positive outside = 0.55, *p *= 0.049; Fig. 5). Although this was only a single cluster, it is noteworthy because it was adjacent to the only breeding site in the community and was at the point of transition from a peri-urban to a rural environment. In addition, outside the cluster in the northeast corner of Fig. 5, the prevalence of *P. falciparum* infection was higher within the ellipse in the northeast quadrant than in the second ellipse in the southwest quadrant of the Fig. (1.41% ± 3.90% *vs.* 0.32% ± 1.38%, *p *= 0.0013). These observations are consistent with a gradient of decreasing prevalence for *P. falciparum* infection with increasing distance from the cluster associated with the breeding site inside the black circle (Fig. 5).

### Household-level factors and high space-time clusters

In an effort to determine whether observed space-time clusters could be explained by household factors (household size, number of women with children in household, recent spraying, recent plastering or painting, and bed net use), the data were modelled using two approaches. With the first approach, the households were classified as occurring either inside or outside of a significant space-time clusters. For each household factor, the frequencies were tabulated according to presence or absence inside a space-time cluster, and Pearson’s Chi square tests were used to determine whether household factors explained the space-time clustering. None of these results were statistically significant after adjusting for multiple testing using a Bonferroni correction approach. Thus, the household factors were not considered to explain the observed space-time clustering using this approach.

Because the results obtained with the analytical approach above could result from modelling the data at the household level (as opposed to the individual level), the second approach employed a random effects model. Here a random effect for individual-level variation in smear positivity, and another random effect for households were incorporated into a hierarchical regression model. Using this model, malaria test result positivity (*P. falciparum* infection at the individual level) was modelled against time period (year and month of malaria test screening), household characteristic (presence or absence of the characteristic) and cluster type (presence or absence inside of a high space-time cluster). The results for these analyses are provided in Additional file 1: Table S1. After correcting for multiple testing using a Bonferroni approach, none of the household-level factors examined were associated with the presence or absence of households within a space-time cluster of high values.

## Discussion

In the studies reported here, spatial, temporal and spatio-temporal clustering of the prevalence of *P. falciparum* infection was examined at rural study sites in The Gambia (Gambissara, Fig. 2) and Mali (Dioro and Dangassa, Figs. 3, 4) and a peri-urban site in Senegal (Madina Fall, Fig. 5). In a spatial sense, the random patterns observed in Mali suggest that households in Dangassa and Dioro may be affected similarly by common environmental factors and their proximity to large numbers of breeding sites. This finding is consistent with earlier studies of *P. falciparum* infection and malaria in Mali [8]. In addition, the prevalence of *P. falciparum* infection at the two Mali sites was consistently higher than those in Gambissara and Madina Fall.

Most housing factors were not associated with clustering, which may be because of consistency in housing and malaria prevention strategies in Dangassa, Dioro and Madina Fall, although not in Gambissara (which had substantial variability in the roofs, eaves and walls of houses at a single site). Although household-level factors differed among study sites, most had little site-to-site variability. For instance, reported bed net use was 88%, 100% and 95% for the Dangassa, Dioro and The Gambia sites, respectively. While proper bed net use tends to be over reported, the consistently high reported bed net use in these communities may suggest that those factors are unlikely to account for spatial or temporal variation in the prevalence of infection as mentioned by Brooker et al. [5]. As noted above, the exception to this generalization was western Gambissara where houses with thatched roofs, open eaves and mud walls had a consistently higher prevalence of *P. falciparum* infection than the nearby community of Gambissara Town where houses had metal roofs, closed eaves with brick walls and a lower prevalence of *P. falciparum* infection (Fig. 3, Table 1).

### Study strengths

This study employed large sample sizes with over 1400 enrolled subjects at each study site. Study participants found to have uncomplicated *P. falciparum* malaria at any site received free treatment and follow-up, to encourage continued participation and retention within the cohort. Study participants diagnosed with severe or complicated *P. falciparum* malaria were transported by ambulance on the day of diagnosis to the nearest hospital where they were treated free of charge and returned home to their community after recovery.

### Potential limitations

This study focused on the household prevalence of *P. falciparum* infection; i.e., on the presence or absence of infection rather than the clinical aspects or severity of malaria. Therefore, it is possible that the inclusion of other factors such as parasite density or other outcomes such as incidence of *P. falciparum* infection could yield different results. Indeed Greenwood [15] has suggested that the clustering of genetically determined red cell abnormalities or immune response genes may also contribute to within-village differences. Moreover, it is possible that the timing of the smear surveys played a role, because the surveys were often limited to a few days or weeks. It is also worth noting that this study used fixed classifications for the dry and rainy seasons at each of the study sites. Although the goal of this study was to capture the beginning and end of the transmission season each year, it was difficult to accurately predict the onset and decline of seasonal rainfall. Sissoko et al. have noted that various lag times may occur in malaria transmission and that humidity and temperature have important effects on malaria transmission [29]. In addition, data on entomologic and environmental factors such as flooded areas and breeding sites were not captured and may also have contributed to the clustering observed. Although this study included household data on the vector control strategies recommended by the World Health Organization (ITNs and Indoor Residual Spraying [IRS]), neither of those strategies was associated with spatial clustering of *P. falciparum* infection or its absence, although it is possible that the within-village homogeneity of these factors influenced their linkage to the clustering of *P. falciparum* infection. The distances between households differed among the sites, which changed the meaning of a household subgroup or cluster from site to site. However, there was no evidence that the distance between households influenced the likelihood that a house would be inside (or outside) a cluster. Finally, another limitation of this study is that it did not examine the incidence of *P. falciparum* malaria and, therefore, could not compare the spatial or temporal distributions of *P. falciparum* infection to those of malarial disease (uncomplicated malaria) which may have different spatial and temporal patterns from asymptomatic *P. falciparum* infection.

## Conclusions

In these studies, spatial clustering of *P. falciparum* infection was found in The Gambia, temporal clustering in Dioro and Dangassa, Mali and spatio-temporal clustering in the peri-urban community of Madina Fall in Senegal.

For example, Smith et al. found that uncomplicated malaria cases clustered near index cases in low prevalence areas [30]. Although the detection of infection strategy described here focused on asymptomatic persons with *P. falciparum* infection, those individuals may still transmit *P. falciparum* parasites, particularly in non-endemic areas [12]. However, because of the high cost of active surveillance, additional efforts should be made to pool data from different sources, including governmental health systems, in order to draw conclusions about larger populations and study areas.

In terms of the hypotheses proposed, note that the analysis of these data identified clustering of *P. falciparum* infection in space (Gambissara), in time (Dangassa and Dioro) and in both time and space (Madina Fall). Those observations also suggest that this information may have the potential to predict/facilitate the effectiveness of control measures such as the restoration of financial support in Dioro, the effects of larviciding and vector control in Madina Fall and the effects of improved housing in Gambissara.

## Supplementary information

**Additional file 1: Table S1.** Random effects analyses assessing relationship between household characteristics and presence/absence of households inside a high space-time cluster.

## Data Availability

The data used or analyzed in this study are available from the corresponding author on reasonable request with approval from the Mali, Gambia, Senegal and Tulane IRBs.

## Abbreviations

ACD: active case detection
ACT: artemisinin combination therapy
GIS: geographic information system
GPS: geographic positioning system
Hb: hemoglobin
ICEMR: International Center of Excellence for Malaria Research
IPTp: intermittent preventive treatment (of malaria) during pregnancy
IRB: Institutional Review Board
LISA: Local Indicators of Spatial Association
LLIN: long-lasting insecticidal net
LMIC: low and middle income country
PCD: passive case detection
SAG: Scientific Advisory Group
WHO: World Health Organization
