# Supplementary material for: Clustering of asymptomatic Plasmodium falciparum infection and the effectiveness of targeted malaria control measures
Source: Malar J. 2020 Jan 21;19:33. doi: 10.1186/s12936-019-3063-9 (PMC6975028; doi:10.1186/s12936-019-3063-9)
Supplement: Supplementary file 1 — Additional file 1: Table S1. Random effects analyses assessing relationship between household characteristics and presence/absence of households inside a high space-time cluster. [file 12936_2019_3063_MOESM1_ESM.docx]

Additional file 1: Table S1.

Random effects analyses assessing relationship between household characteristics and presence/absence of households inside a high space-time cluster.

| **Site/Household characteristic** | **Dry Season** | | | **Rainy Season** | | |
| --- | --- | --- | --- | --- | --- | --- |
|  | **OR** | ***p^1^*** | ***p^2^*** | **OR** | ***p*^1^** | ***p*^2^** |
| **Dangassa** |  |  |  |  |  |  |
| Household size > 5 | 1.37 (1.01, 1.86) | .045 | .910 | 1.35 (0.92, 1.96) | .118 | .420 |
| >2 mothers with children < 5 yrs | 1.04 (0.62, 1.75) | .883 | .592 | 1.13 (0.64, 2.01) | .666 | .751 |
| Recent spraying | 0.90 (0.64, 1.26) | .530 | .746 | 1.17 (0.57, 2.42) | .671 | .711 |
| Recent plastering/painting | 2.01 (1.16, 3.49) | .013 | .112 | 0.84 (0.49, 1.43) | .525 | .382 |
| Use of mosquito nets | 0.93 (0.58, 1.48) | .753 | .093 | 0.53 (0.28, 1.02) | .057 | .510 |
| **Dioro** |  |  |  |  |  |  |
| Household size > 5 | 0.87 (0.66, 1.14) | .306 | .019 | 0.96 (0.72, 1.27) | .759 | .188 |
| > 2 mothers with children < 5 yrs | 0.69 (0.41, 1.17) | .166 | .669 | 1.13 (0.75, 1.69) | .563 | .238 |
| Recent spraying | 0.93 (0.67, 1.29) | .660 | .846 | 1.02 (0.75, 1.38) | .912 | .462 |
| Recent plastering/painting | 0.64 (0.29, 1.39) | .257 | .104 | 0.99 (0.44, 2.24) | .976 | .199 |
| Use of mosquito nets | 1.37 (0.95, 1.98) | — | — | 1.20 (0.78, 1.85) | — | — |
| **The Gambia** |  |  |  |  |  |  |
| Household size > 5 | — | — | — | — | — | — |
| > 2 mothers with children < 5 yrs | 0.56 (0.35, 0.92) | .021 | — | 0.37 (0.19, 0.74) | .005 | — |
| Recent spraying | — | — | — | — | — | — |
| Recent plastering/painting | — | — | — | — | — | — |
| Use of mosquito nets | — | — | — | — | — | — |
| **Senegal** |  |  |  |  |  |  |
| Household size > 5 | 0.54 (0.10, 2,88) | .468 | .632 | — | — | — |
| > 2 mothers with children < 5 yrs | — | — | — | — | — | — |
| Recent spraying | — | — | — | — | — | — |
| Recent plastering/painting | — | .301 | — | — | — | — |
| Use of mosquito nets | 2.23 (0.53, 9.39) | .274 | .750 | — | — | — |
| *Note*. Analyses carried out using a random effects model for modelling malaria test result positivity against time period, household characteristic (presence or absence), and cluster type (presence or absence inside of a high space-time cluster). OR = Odds ratio; — = insufficient variation for calculating p value  p^1^ = p-value for comparing presence of household characteristic with test result positivity  p^2^ = p-value testing the interaction between presence of household characteristic (presence or absence), and presence of a household inside of a high cluster (presence or absence)  ^*^Significant at the 5% significance level. Type I error = .01 to account for multiple testing within each group of household factors by study site. | | | | | | |
